# Correction: A Single-Arm Phase 2 Trial of Doxorubicin Plus Zalifrelimab (Anti–CTLA-4 Antibody) and Balstilimab (Anti–PD-1 Antibody) in Advanced/Metastatic Soft Tissue Sarcomas

**DOI:** 10.1158/1078-0432.CCR-26-1903

**Published:** 2026-07-17

**Authors:** Breelyn A. Wilky, Katherine A. Julian, Alessandra Maleddu, Anne C. Mailhot, Chelsey R. Cartwright, Dexiang Gao, Cristiam Moreno Tellez, Lindsey E. Kemp, Nicholas R. Therrien, Sumra S. Chaudhry, Jeffrey D. Rytlewski, Qierra R. Brockman, Eduardo Davila, Anthony D. Elias

In the original version of this article (1), Supplementary Table S2 was incorrect: Patient 12 was erroneously marked as deceased. Supplementary Table S2 has been replaced with a corrected version in the latest online HTML version of the article. The authors regret the error.
